# Sperm structure and motility in the eusocial naked mole-rat, *Heterocephalus glaber*: a case of degenerative orthogenesis in the absence of sperm competition?

**DOI:** 10.1186/1471-2148-11-351

**Published:** 2011-12-05

**Authors:** Gerhard van der Horst, Liana Maree, Sanet H Kotzé, M Justin O'Riain

**Affiliations:** 1 Department of Medical Bioscience, University of the Western Cape, Bellville, South Africa; 2 Division of Anatomy and Histology, Department of Biomedical Sciences, Stellenbosch University, Parow, South Africa; 3 Department of Zoology, University of Cape Town, Cape Town, South Africa

## Abstract

**Background:**

We have studied sperm structure and motility in a eusocial rodent where reproduction is typically restricted to a single male and behaviourally dominant queen. Males rarely compete for access to the queen during her estrus cycle, suggesting little or no role for sperm competition.

**Results:**

Our results revealed an atypical mammalian sperm structure with spermatozoa from breeding, subordinate and disperser males being degenerate and almost completely lacking a "mammalian phylogenetic stamp". Sperm structure is characterized by extreme polymorphism with most spermatozoa classified as abnormal. Sperm head shapes include round, oval, elongated, lobed, asymmetrical and amorphous. At the ultrastructural level, the sperm head contains condensed to granular chromatin with large open spaces between the chromatin. Nuclear chromatin seems disorganized since chromatin condensation is irregular and extremely inconsistent. The acrosome forms a cap (ca 35%) over the anterior part of the head. A well defined nuclear fossa and neck with five minor sets of banded protein structures are present. The midpiece is poorly organized and contains only 5 to 7 round to oval mitochondria. The flagellar pattern is 9+9+2. A distinct degenerative feature of the tail principal piece is the absence of the fibrous sheath. Only 7% motile spermatozoa were observed which had exceptionally slow swimming speeds.

**Conclusion:**

In this species, sperm form has simplified and degenerated in many aspects and represents a specialised form of degenerative orthogenesis at the cellular level.

## Background

Sperm competition is the norm in the animal kingdom and in many taxa, such as amphibians, snakes, passerine birds and mammals [1-3], sperm structure and function can be correlated with the level of sperm competition [3-7]. Gomendio and Roldan [6] found that in promiscuous species of primates and muroid rodents which experience a high degree of sperm competition, there tends to be an increase in sperm length compared to species where there is less sperm competition. A subsequent analysis of 100 species of rodents supported this latter conclusion [8] and Anderson and Dixson [9] have clearly found a positive correlation between volume of the sperm midpiece and sperm competition. Tourmente et al. [3] showed that an increase in the level of sperm competition in snakes is correlated with an increase in sperm length and that this elongation is largely explained by increases in midpiece length. In snakes, the midpiece contains structures which, in other taxa, are present in the remainder of the flagellum, suggesting that it may integrate some of its functions. Pitnick et al. [10], however, cautioned in a review that several studies have shown no relationship in terms of sperm length and sperm competition in mammals [11-13].

Spermatozoa with more rapid swimming speeds have a fertilizing advantage during sperm competition in the Atlantic salmon [14], mallards [15], domestic poultry [16] and mammals [7] as evaluated by means of computer aided semen analysis (CASA). Anderson et al. [17] showed that the mitochondrial membrane potential was not only higher but better maintained in chimpanzee spermatozoa (high sperm competition) compared to human spermatozoa (low sperm competition). Maree [18] produced similar results when comparing sperm mitochondrial membrane potential in humans and three species of Old World monkeys. Apart from these investigations on sperm function and those on sperm morphometry mentioned above, no studies on sperm competition in mammals have included data on potential differences at the sperm ultrastructural level. Moreover, few studies have compared the structure and function of spermatozoa in promiscuous versus eusocial mammals (monogamous), largely because the latter mating strategy is so rare.

Naked mole-rats (*Heterocephalus glaber*) are one of only two eusocial mammals [19-21] with reproduction typically restricted to a single female (queen) and male within large colonies of 40-90 individuals [19,20,22]. Although not strictly monogamous, as multi-paternity has been recorded for this species [23], most other males and all other females (subordinates) are reproductively suppressed. This restriction of breeding to a small subset of the entire population (queen and 1-3 breeding males) possibly presents a low risk for sperm competition and it is predicted to have shaped the structure (simple) and motility (slow) of spermatozoa in this species.

Here we describe the sperm structure of breeding, subordinate and disperser male naked mole-rats using light and electron microscopy (scanning and transmission). The sperm motility of these naked mole-rats has been studied by means of CASA to establish baseline parameters. This data was used to test the hypothesis that levels of male intrasexual competition may influence the structure and motility of spermatozoa. We predict that in naked mole-rats with limited intrasexual competition amongst males, sperm head length, midpiece length and tail length will be shorter when compared to promiscuous species. It is further predicted that breeders will have better sperm quality than subordinates since the latter are reproductively suppressed.

## Results

### Sperm structure: Light microscopy

**Figure 1:**
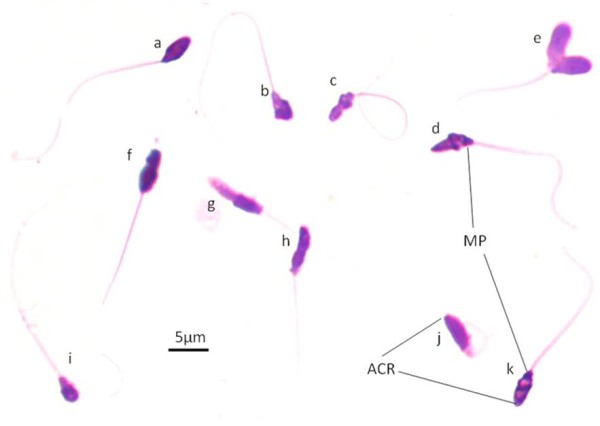
**Bright field microscopy of naked mole-rat spermatozoa stained with SpermBlue and showing evident sperm polymorphism**. a) normal spermatozoon; b) compressed head; c) lobed head and curled tail; d) cone-shaped head; e) double macro-heads; f-h) multi-lobed elongated heads; g) head without acrosome; i) micro-head; j) amorphous head; k) apparent nuclear vacuoles representative of fragmentation. MP = midpiece, ACR = acrosome.

A typical naked mole-rat spermatozoon is characterized by an irregular shaped head, a neck, a poorly defined midpiece and a tail. The most striking feature emerging from the micrographs is the large amount of polymorphism encountered, particularly in terms of sperm head shape (Figure 1). Consequently, sperm structure varies markedly with sperm head shape, including round, oval, elongated, slightly lobed, severely lobed and asymmetrical heads. There are several further variations that could only be described as irregular or amorphous (Figure 1). Due to this variation in sperm head shape, it was difficult to statistically determine differences between the breeders, subordinates and dispersers in terms of sperm morphology. However, there did not appear to be any striking difference in the type of sperm head abnormalities encountered in these three groups of males and no significant differences (p > 0.05) were found in their sperm morphometry. Jointly, the basic morphometric dimensions such as length, width and perimeter (Table 1) indicate that the naked mole-rat has very small spermatozoa relative to other mammals.

**Table 1:** Sperm morphometry parameter measurements* (average ± SD) of the sperm head, midpiece and tail

Parameter	Measurement
Head:	
Length (μm)	3.98 ± 0.35
Width (μm)	2.25 ± 0.15
Area (μm^2^)	7.47 ± 0.92
Perimeter (μm)	11.52 ± 0.81
Ellipticity	4.27 ± 0.35
Elongation	0.27 ± 0.04
Midpiece:	
Length (μm)	1.09 ± 0.10
Width (μm)	1.09 ± 0.11
Area (μm^2^)	1.10 ± 0.19
Perimeter (μm)	4.25 ± 0.38
Tail:	
Length (μm)	28.06 ± 3.13

*Data are combined for breeders, subordinates and dispersers.

Light microscopy of the midpiece revealed a very small and irregularly shaped structure (length and width approximately 1.09 μm). The midpiece closely adheres to the sperm head and often exhibits irregular borders. It was accordingly sometimes difficult to distinguish the midpiece from the posterior part of the head, particularly the neck. The average tail length is 28.06 μm (SD ± 3.13) and the tail appears to have an even diameter throughout. Although the end piece of the tail is not well defined and apparently short, there appears to be very few tail abnormalities.

### Sperm structure: Scanning and transmission electron microscopy

There were no clear ultrastructural differences among spermatozoa from the cauda epididymis, vas deferens or ampulla.

### Sperm head

**Figure 2:**
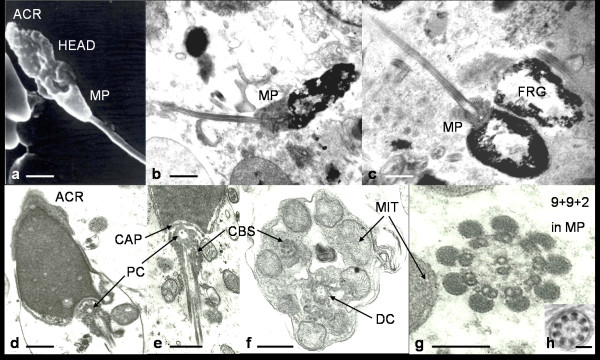
**Scanning and transmission electron micrographs of naked mole-rat spermatozoa**. a) Scanning electron micrograph of a typical multi-lobed sperm head, b-h) Transmission electron micrographs of naked mole-rat spermatozoa sectioned in different planes: b) Longitudinal section almost similar to spermaozoon in (a) showing irregular arranged chromatin, small midpiece and thin tail; c) Two sperm heads showing severe fragmentation and small midpiece; d) Sperm head containing a simple acrosomal cap; d-e) Proximal centriole with capitulum and striations of cross banded structures; f) Transverse to oblique section of anterior part of midpiece showing mitochondria with tubular-like cristae, some cross banded structures and distal centriole; g) Axoneme (9+2 microtubules) surrounded by nine outer dense fibres in midpiece; h) Only axonome in tail principal piece without a fibrous sheath. ACR = acrosome, HEAD = sperm head, FRG = fragmented sperm head, CAP = capitulum, PC = proximal centriole, CBS = cross banded structures, DC = distal centriole, MIT = mitochondria, MP = midpiece. Scale bars: a, b, c = 1 μm; d, e, f = 0.5 μm; g, h = 0.25 μm.

Figure 2a represents a typical multi-lobed sperm head as viewed by scanning electron microscopy (SEM). The surface morphology reveals an irregular sperm head and small midpiece. The acrosome is poorly defined and difficult to discern by SEM.

Figures 2b-h are transmission electron micrographs depicting the details of the different components of the spermatozoa. Figure 2b shows all the major components of a naked mole-rat spermatozoon in longitudinal section. In this figure the head is multi-lobed, the midpiece is small and the tail is homogenously thin. The head consists of granular chromatin that is not fully condensed and in almost all spermatozoa large intra-nuclear spaces are evident which appears to be dispersed chromatin. Figure 2c presents two sperm heads that appears severely fragmented. Figure 2d shows a very simple acrosome (acrososmal cap) that covers 30-40% of the head area.

### Neck

The basal plate is connected to the nucleus by means of longitudinal satellite fibres. While the nuclear fossa is well defined, the capitulum, an electron dense structure, is poorly developed. There are two dominant and five smaller cross banded structures emerging from the capitulum and running longitudinally towards the midpiece (Figures 2d and 2e). The two dominant cross banded structures each forms two additional cross banded structures lower down in the neck/midpiece. There are accordingly a total of nine cross banded structures (each exhibiting about 12 cross striations) that eventually connect with the outer nine dense fibres surrounding the 9+2 microtubules (axoneme) (Figures 2d-f). These nine cross banded structures furthermore connect with the outer nine fibres close to the distal centriole. Just below the capitulum and surrounded by the cross banded structures is a clearly demarcated proximal centriole (Figures 2d and 2e) which is 90° orientated in terms of its central axis to the distal centriole. The distal centriole (Figure 2f) gives rise to the axoneme, which typically has the 9+2 microtubule arrangement.

### Midpiece

The shape of the midpiece varies greatly and both SEM (Figure 2a) and TEM (Figures 2b-f) confirmed the light microscopic observations (Figure 1). In both transverse and longitudinal sections, the irregularity of the midpiece is demonstrated. There appears to be five to seven mitochondria present which reveal two major forms. The one form is elongated and the other oval to spherical. The cristae mitochondriales have a spherical or wavy form (Figures 2d-f) and conform to the orthodox state. In the midpiece, the 9+9+2 pattern of the axoneme and the outer dense fibres can be seen (Figure 2g). The nine outer dense fibres approximately have the same diameter. Other structures in the midpiece include various vesicles of different size and shape (Figure 2e). These may represent some of the byproducts of spermiogenesis and are apparently not discarded as part of the contents of the cytoplasmic droplet.

### Tail

There is not a defined annulus demarcating the posterior part of the sperm midpiece and the start of the principal piece of the tail. The principal piece of the tail contains the 9+9+2 axonemal-outer dense fibre configuration as in the midpiece and shown in Figures 2f and 2g. Surprisingly, there is no outer fibrous sheath incorporating dense fibres three and eight to form two longitudinal columns. Consequently, the ribs of the fibrous sheath in the principal piece connecting the longitudinal columns are also lacking in naked mole-rat spermatozoa (Figure 2h). Towards the end of the tail, the outer dense fibres are closely associated with the outer doublets of the axoneme. The end piece is not clearly demarcated but only has the axoneme (no dense fibres) and no additional fibres on its outside.

### Sperm concentration and sperm motility

Sperm concentration varied from as little as 5 × 10^6^/ml to about 100 × 10^6^/ml with an average of 43.0 × 10^6^/ml (SD ± 45.2) (Table 2). No significant differences (p > 0.05) were found in the sperm concentration of breeders, subordinates and dispersers. The volume of the fluid within each ampulla was approximately 5 μl and accordingly the maximum number of spermatozoa in both ampullae was estimated in the region of about 1 million spermatozoa and at least 50 000 when the queen was in estrus. However, it was difficult to determine sperm concentration accurately due to the presence of abundant vesicles within the semen that were slightly larger than the sperm heads. Thus, these mentioned values represent maximum estimates for sperm concentration.

**Table 2:** Sperm kinematic parameter measurements* (average ± SD) captured at 50 frames/second

Parameter	Measurement
Motility (%)	7.3 ± 6.7
Concentration (×10^6^/ml)	43.0 ± 45.2
VCL (μm/s)	35.5 ± 6.7
VSL (μm/s)	16.3 ± 6.3
VAP (μm/s)	26.1 ± 5.9
LIN (%)	44.4 ± 9.5
STR (%)	60.6 ± 10.8
WOB (%)	73.0 ± 4.4
ALH (μm)	0.5 ± 0.4
BCF (Hz)	4.9 ± 5.3

*Data are combined for breeders, subordinates and dispersers.

VCL = curvilinear velocity, VSL = straight-line velocity, VAP = average path velocity, LIN = linearity, STR = straightness, WOB = wobble, ALH = amplitude of lateral head displacement, BCF = beat cross frequency.

**Figure 3:**
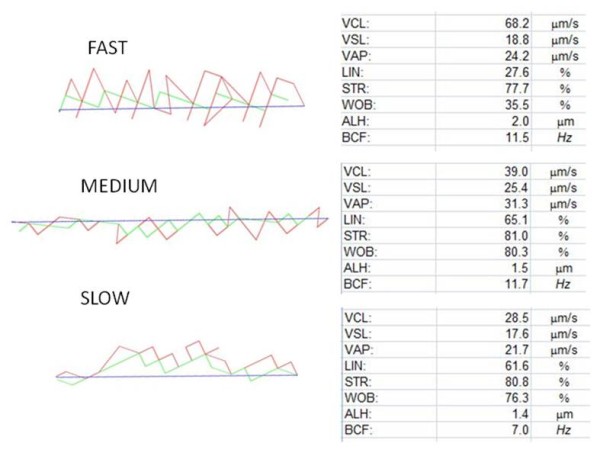
**Three representative sperm motility patterns of naked mole-rat spermatozoa recorded at 50 frames/second**. Red line = VCL, blue line = VSL and green line = VAP. The kinematic data for each track is shown on its immediate right (cut off values based on VCL (μm/s) = Fast > 45 > Medium > 35 Slow). VCL = curvilinear velocity, VSL = straight-line velocity, VAP = average path velocity, LIN = linearity, STR = straightness, WOB = wobble, ALH = amplitude of lateral head displacement, BCF = beat cross frequency.

Table 2 shows the combined sperm motility data for the fifteen males since there was no significant differences (p > 0.05) found among the three groups (breeders, subordinates and dispersers). The total percentage sperm motility was low and varied from 1-15% (average 7.3% SD ± 6.7). The average kinematic parameters of these spermatozoa were representative of slow swimming sperm (VCL = 35.5 μm/s SD ± 6.7) with fairly good progression (STR = 60.6% SD ± 10.8) and low linearity (LIN = 44.4% SD ± 9.5). However, the range for VCL varied between 15-68 μm/s. It appeared that the faster the spermatozoa swim, the greater was the amplitude of lateral head displacement (ALH). The overall effect was that fast swimming spermatozoa had a lower linearity than slow swimming spermatozoa but the fast spermatozoa swim more vigorously (large head and tail oscillations). Figures 3a-c depict representative examples of the motility patterns and kinematic parameters of fast, medium and slow moving spermatozoa among this characteristic "slow" swimming population. Furthermore, the fast swimming spermatozoa represented 0-1% of all motile sperm, the medium swimming spermatozoa 3-5% and the slow swimming population 93-96%. Hence, the low average VCL of 35 μm/s can be explained by the fact that the majority of motile spermatozoa had a low VCL and accordingly swim sluggishly.

## Discussion

A typical mammalian spermatozoon consists of a head partly covered by an acrosome, a neck and a flagellar-like tail. The head of the mammalian spermatozoon is ovate, ensiform or falciform and dorsoventrally flattened. The neck typically consists of the connecting piece and the centriole [24]. The mammalian sperm tail contains an axonemal complex of microtubules and a further nine outer dense fibers to complete the 9+9+2 pattern [25]. In the midpiece of the mammalian spermatozoon, the axoneme and outer dense fibers are enclosed by a long sheath of mitochondria. The mitochondria itself are elongated and arranged around the core of the sperm tail in a helical fashion. The number of mitochondrial gyres varies between mammalian species, with the relatively short midpiece of the human consisting of about 15 gyres, whereas the exceptionally long midpiece of several rodent species contain as many as 300 gyres [24]. In the principal piece of the flagellum, the axonemal-outer dense fiber complex is surrounded by the fibrous sheath, which is divided into several transverse ribs along the length of the principal piece [26].

The spermatozoa of naked mole-rats in this study deviate markedly from that of virtually all mammals. The sperm head surface is extremely irregular and often form small or large lobes with a high percentage of either dispersed chromatin or so-called nuclear vacuoles. The lobed nucleus in particular appears to be degenerate compared to that of most mammals. Together these morphological attributes would result in most naked mole-rat spermatozoa being classified as "abnormal". Importantly, these attributes are not considered to be a major consequence of inbreeding as the individuals used in this study originate from colonies in a captive population that include both inbred and outbred pedigrees and a low mean level of inbreeding (F = 0.163) [27]. The high inbreeding coefficient reported in a previous study (F = 0.45) among four wild-caught colonies of naked mole-rats in Kenya [28], can be due to the fact that three of these colonies were collected within 5 km of each other. New colonies of naked mole-rats are usually formed by fissioning and thus neighbouring colonies could have a recent common maternal ancestor [23,29].

The neck of the naked mole-rat spermatozoon contains a poorly developed capitulum which gives rise to five banded columns. In most mammals and particularly in rodents, however, the capitulum represents a large, solid and well developed structure [26]. The well defined midpiece of most mammalian species, particularly in terms of the highly organized helical/non-helical arrangement of mitochondria, is replaced in the naked mole-rat by a small and generally disorganized midpiece. The midpiece length is the shortest of all mammals so far recorded (± 1 μm) [30] and the total number of mitochondria (± 7) is also among the lowest for any mammalian species [26]. Furthermore, the mitochondria are randomly dispersed and their form varies even within the same sperm midpiece. Accordingly, the midpiece of naked mole-rat spermatozoa appears to show various degenerate features.

The greatest deviation from the mammalian pattern in the naked mole-rat spermatozoon is the structure of the principal piece of the sperm tail. In this species, there is no apparent difference found in the size of the nine outer dense fibers surrounding the axoneme. However, in many mammalian species the outer dense fibers numbered 1, 5 and 6 are distinctly larger than the other six fibers and some species also have a larger fiber in position 9 [26]. Although the 9+9+2 pattern persists in naked mole-rat spermatozoa, there is no fibrous sheath present. One of the main suggested functions for the fibrous sheath is to provide structural support/strength to the tail beating rapidly in a viscous medium as encountered in the female reproductive tract [26,31]. Structurally these deviations in the principal piece thus represent further simplified and possibly degenerative features of the naked mole-rat spermatozoon.

Sperm structure has been extensively used as a tool to assist in both taxonomic and phylogenetic studies and more recently as an indicator of relative sperm competition. For example, van der Horst et al. [32] showed that acrosome structure and shape can be used to distinguish among four very closely related ferret species. Breed [33,34] has furthermore shown that sperm head structure is related to phylogenetic relationships in rodents in addition to their phylogenetic derivation (primitive versus advanced structures). However, despite the fact that certain species' spermatozoa may be derived or have become more specialized or simplified, one seldom encounters the situation where there is such a large variability in sperm form within a given species as observed in the naked mole-rat. Human sperm provides a rare example of sperm polymorphism and in human clinical spermatology, any deviation in sperm structure from normal is defined as abnormal according to the so called Strict Criteria [35]. Thus, in this study we revealed that, similar to humans, naked mole-rat spermatozoa have a high degree of polymorphism.

An important question that emerges is: which of these "polymorphic" spermatozoa are normal or abnormal and how does sperm morphology affect their ability to fertilize an oocyte? During standard semen analysis procedures, the normality of sperm morphology is an important characteristic in determining the fertilizing potential of spermatozoa [35,36]. In many mammalian species (natural populations) a relatively high percentage of spermatozoa in the ejaculate are morphologically normal (> 80%) [37]. In most of these species the level of sperm competition is high and it can be assumed that there is strong selection pressure to produce a high percentage of spermatozoa that are structurally and functionally normal [38]. The end result is that most spermatozoa have an almost equal chance of fertilizing an oocyte.

Previous studies which mentioned the existence of variation in male fertility of some mammalian species, still reported a relatively high percentage of morphologically normal sperm, e.g. 77% (range 12-97%) in natural populations of red deer [39] and 76% (range 6-91%) in adult dogs [40]. Even the endangered black-footed ferret (*Mustela nigripes*), which is exposed to a high degree of inbreeding, had 68% normal spermatozoa in the breeding season [41]. Interestingly, in humans who typically have a low risk of sperm competition, males with more than 15% normal spermatozoa is regarded as fertile according to Strict Criteria [42] and the lower reference limit for normal forms is 4% [35]. Preliminary results from our laboratory have shown that the naked mole-rat has only few normal spermatozoa (± 5-15%, data not shown). Thus, most of the polymorphic spermatozoa in humans and naked mole-rats are apparently abnormal and accordingly not variations of normal spermatozoa.

Consequently, sperm competition would appear to be extremely unlikely in naked mole-rats and there seems to be no need to produce perfectly formed and highly motile sperm. Parker [43] emphasized this principle by mentioning that the production of high quality, error free spermatozoa is costly and that there will be selection against it if the costs are not equal to or outweighed by the benefits (fertilizing the oocyte). Thus, in the absence of sperm competition, there may be little benefit in investing energy on the quality of sperm production [44]. However, when there is a high risk of sperm competition, every sperm counts and selection will favour the production of high quality spermatozoa [10]. Further evidence for the absence of sperm competition in the naked mole-rat is entrenched in its sperm structure. Both the short midpiece and short tail (± 28 μm) of naked mole-rat spermatozoa is typical of mammals with a low risk of sperm competition [8,9,45]. The possible effect of a lack of sperm competition on the size and structure of spermatozoa is indirectly emphasized by Lijfeld et al. [46] who reported that an increased risk of sperm competition selects for longer spermatozoa and reduces between-male and within-male variation in the sperm length in passerine birds. However, another factor contributing to the small size of naked mole-rat spermatozoa could be this species' lower metabolic rate [47]. Recent studies on the effect of metabolic rate on sperm size [48,49] have shown that there is a positive relationship between the mass-specific metabolic rate and the total sperm length of both eutherian and marsupial mammals and that species with a lower mass-specific metabolic rate produce uniformly small spermatozoa [48,49].

Despite the fact that few of the naked mole-rat spermatozoa are structurally and physiologically normal (e.g. motile), the breeding males in this study were all producing healthy litters of pups prior to their removal. This suggests that they are capable of producing sufficient normal spermatozoa available to fertilize multiple oocytes. The relatively high average sperm concentration found in the current study was probably due to the fact that a very high sperm concentration (100 × 10^6^/ml) was measured in only one male and therefore skewed the data. However, for most males the sperm concentration varied between 5-50 × 10^6^/ml and the lower limit of the current study is comparable to the 1.8-8.6 × 10^6 ^spermatozoa in one half of naked mole-rat reproductive tract previously reported by Faulkes et al. [50]. This low average concentration of spermatozoa in the naked mole-rat could be another effect of the absence of sperm competition and is consistent with the theory that an increase in sperm competition will increase the number of sperm produced by a male [4]. An extreme case of this phenomena is found in the yellow seahorse (*Hippocampus kuda*), a species which also lacks sperm competition, where the testes only contain about 300 spermatozoa [51] and results in a sperm:egg ratio comparable to that of the social insects [52].

The degenerative structural state of both the midpiece and the tail could possibly explain the poor motility of naked mole-rat spermatozoa. The kinematic parameters clearly showed that there was both low percentage motility as well as sluggish moving spermatozoa. The low percentage motile spermatozoa recorded in the current study (7.3%) were similar to the 5% motile spermatozoa reported by TB Hildebrandt [personal communication]. Although Faulkes et al. [50] also found relatively low percentage motile spermatozoa in naked mole-rats (< 50%), they reported a significantly lower sperm concentration and lower percentage motile spermatozoa in subordinates relative to breeders, which were not evident from the current study. The spermatozoa in the current study were swimming at an average curvilinear velocity of 35 μm/s, which may well be the lowest recorded for any mammalian species. In other social mole-rats of the same family (Bathyergidae), the average sperm velocity is 148 μm/s and a high percentage of sperm motility is evident [53]. Even humans, who have a high percentage of abnormal spermatozoa, typically have more than 60% motile sperm and they swim with an average velocity of about 90-120 μm/s [18,54,55]. The slow swimming speed of naked mole-rat spermatozoa could thus be the result of both the short tail, which beats with a lower force, and the small midpiece with few mitochondria, which may generate less energy for motility.

Another aspect that requires attention is how much simplification or degeneration is present in naked mole-rat spermatozoa? Part of the answer may be found by looking at sperm structure in the monotremes such as the platypus. Here typical mammalian sperm features are maintained and the fibrous sheath of the principal piece of the tail is well developed [56]. In contrast, marsupial spermatozoa seem to share sperm characteristics with the sauropsids rather than mammals and therefore reflect a more primitive condition. However, even in this instance the fibrous sheath is a typical feature of the principal piece of the tail. The absence of this feature in naked mole-rat spermatozoa when compared to the primitive mammals accordingly supports the view that this is a degenerative feature in naked mole-rats and not a primitive or simplified feature. Naked mole-rat spermatozoa seem to be derived from ancestral rodent sperm with a hooked acrosome. Breed [34] concluded in his study on rodent spermatozoa that, "as the hook-shaped sperm head and long sperm tail occur across the muroid subfamilies, as well as in the heteromyid rodents, it is likely to be the ancestral condition within each of the subfamilies with the various forms of non-hooked sperm heads, that are sometimes associated with short tails, being highly derived states". The low number and disorganized nature of the mitochondria in the midpiece of naked mole-rat spermatozoa also appears to show degenerate features rather than simplification. When a spermatozoon has simplified, there is usually great order in terms of its organization (e.g. teleost sperm) and contrasts sharply with the situation in naked mole-rats.

We evoke the term 'degenerative orthogenesis' [57] to describe the degenerate appearance and poor motility of naked mole rat spermatozoa. According to Gould [57] (also [58] and [59]), Wilhelm Haacke devised the word "orthogenesis" which means "straight (line) generation" and subsequently "orthogenesis denotes the claim that evolution proceeds along defined and restricted pathways" [57]. In this context Gould [57] based his interpretation on dissecting the work of some eminent evolutionists of their time [58,60-63]. While it is considered as a formalist theory standing against the central Darwinian principle, it has been interpreted in a broader context by many [64,65]. It is particularly Gould [57] that supports a modern use of processes/concepts generally described as saltations (discontinuous evolution, constraints) and channels (internally generated pathways) and includes orthogenesis to understand evolutionary change within the Darwinian framework. These notions above represent two sides of Gould's conviction that the internal structure of an organism can set and constrain the pathways of change [57].

Degenerate animals often have a simpler anatomy than primitive and non-degenerate animals, such as in *Lepas *[65]. De Villiers [65] furthermore emphasized that it is not only the individual animal of a species that is sensitive to stimuli from the environment, but also the embryo and larvae. For example, de Villiers [65] indicated that, in vertebrate embryos, there appears to be a delayed development of certain openings and tracts due to the pressure of assimilated yolk inherited from their ancestors. Accordingly gametes would also be exposed and respond to various stimuli and undergo changes. However, the authors, in agreement with de Villiers [65], do not suggest that all these changes are palingenetic but rather kenogenetic. Morphological degeneration is not a new concept and Eimer [62] referred to this as an environmental impetus of a balance between internal and external forces. However, it was viewed in a narrow formalist context which was difficult to analyze scientifically [66] and therefore required interpretation in a broader framework.

Most structures in naked mole-rat spermatozoa clearly became degenerate, such as components of the head, midpiece, neck and rest of the flagellum. It is important to draw a clear distinction between sperm degenerative features due to inbreeding and those due to the absence of sperm competition. Pure inbreeding degeneration in sperm structure may partly include features such as sperm DNA fragmentation [67] and sperm morphology abnormalities (abnormal size and shape of the head, midpiece and tail) [67-70]. Degenerative changes due to virtually no sperm competition, however, involve a vast simplification in features, for example the absence of the fibrous sheath in the principal piece of the tail (a fundamental mammalian sperm structural feature [26]), an abbreviated midpiece with few simplified mitochondria and a poorly developed capitulum in the sperm neck. To our knowledge, this is the first study to describe the presence of such "degenerative features" in a mammalian spermatozoon.

Hence, in naked mole-rat spermatozoa it appears both inbreeding and the absence of sperm competition may have contributed to abnormal sperm features but that the degenerative features mentioned above represent very specific absence or modification of structures such as the midpiece and tail. It is possible that natural selection forces operated, but that simplification in sperm structure was primarily driven by the lack of sperm competition. This apparent absence of sperm competition was followed by a morphological degeneration of sperm structures, representing a process of degenerative orthogenesis, and is largely based on their reaction to the internal environment. There does not appear to be any advantage or adaptation in this degeneration of sperm structures and the spermatozoa simplified or degenerated to such an extent that it is on a path of no return. In this investigation our interpretation is in line with Gould [57] who considers these older formalist concepts in a broader context in assisting to understand the theoretical base of evolution within a Darwinian framework. Furthermore, our research presents a unique finding that evolutionary processes such as degenerative orthogenesis may occur right up to the cellular level and not only in the individual or embryo as was previously shown.

## Conclusions

It is hypothesized that naked mole-rat spermatozoa have evolved in response to a lack of sperm competition amongst males who are selected for mating by a behaviourally omnipotent queen. Consequently, there was limited selection pressure on spermatozoa and hence they became degenerative. It is surprising that despite the degenerative features and reduced sperm motility, these spermatozoa are nevertheless capable of fertilizing many ova [71] (up to 27 pups in a litter [72]). It is possible that selection pressures in the female to produce a large number of high quality oocytes may compensate for the poor sperm quality. In addition, the oocyte may be specialized in mechanisms that select for the best spermatozoa and may represent sperm selection at the level of female cryptic choice as suggested by Snook [73]. If our hypothesis is accepted, it will imply a balance between developmental facets being selected for in terms of a "limit" to poor sperm quality (degenerative orthogenesis) *versus *developmental pressure for the selection of not only high quality oocytes but also oocytes which can select for the best quality spermatozoa.

## Methods

### Animals used

The study population, initiated with wild-caught founders from various localities in Kenya, has been maintained since 1981 in custom built facilities at the University of Cape Town, South Africa. Husbandry details have been described previously by Jarvis [72]. A total of 15 male naked mole-rats (*Heterocephalus glaber*) were used in this study, including 5 breeders, 5 subordinates and 5 dispersers. Breeders were adult males that regularly consorted (naso-anal grooming) with the queen and were observed copulating with the queen during the estrus period. Subordinate males were also adult males but they were never observed to consort or copulate with the queen. Dispersers were subordinate males in the colony that had strong dispersal tendencies and if presented with foreign conspecifics would consort readily with them [74]. Pedigrees have been constructed for all individuals in this captive population [27] and inbreeding coefficients for the individuals, sourced from 10 captive colonies, ranged from *F *= 0 (outbred, dam and sire from geographically disparate parts of Kenya) through to *F *= 0.5 (highly inbred, inbreeding between siblings) with a mean *F *= 0.163 ± 0.158 SD. The queens that were mated by the five breeding males in this study all produced healthy, viable offspring with the last litter produced prior to removal of the males having a mean size of 10.2 ± 0.8 pups.

Ethical clearance for the study was obtained from both the University of Cape Town (2005/V7/JOR) and the University of the Western Cape (ScR1RC2007/3/30).

### Collection and staining of spermatozoa

Animals were removed from their burrow system and anaesthetized with halothane by putting a mask over the head. Surgical anaesthesia was attained within five minutes. The entire reproductive system was dissected out and put into Ham's F10 medium (Invitrogen, Cape Town, South Africa) at 28°C (to coincide with body temperature of naked mole-rats). This lower temperature margin of the Ham's F10 medium did not have an influence on the pH of the medium (pH remained between 7.6-7.7). Spermatozoa were obtained from the cauda epididymis, vas deferens and enlarged ampulla. Sperm smears were stained with SpermBlue (Microptic S.L., Barcelona, Spain) according to van der Horst and Maree [75] and Maree et al. [76]. A Nikon E50i microscope (IMP, Cape Town, South Africa) fitted with a 100 × oil immersion objective was used to observe the sperm smears and spermatozoa were photographed with a digital fire wire Basler 312fc colour camera (Microptic S.L., Barcelona, Spain). Images were captured with the Cell counter module of the Sperm Class Analyzer (SCA) version 4.1 (Microptic S.L., Barcelona, Spain). Detailed measurements of the different sperm components (head, midpiece, tail) were performed using the image analysis system analySIS FIVE (Wirsam, Cape Town, South Africa). In this instance, a high resolution camera (Olympus Astra 20) fitted onto a Zeiss Photomicroscope III (Zeiss, Cape Town, South Africa) and a 100 × oil immersion objective were used.

### Scanning and electron microscopy

Representative small pieces of epididymis, vas deferens and ampulla tissue were fixed in 2.5% phosphate buffered glutaraldehyde and 1% osmium tetroxide in phosphate buffer. The material was subsequently routinely processed for scanning and transmission electron microscopy (TEM). For scanning electron microscopy (SEM), tissue was dehydrated with an alcohol series and then dried using the critical point drying method, coated with gold and viewed using a Hitachi X650 40 kV scanning electron microscope (Protea Technologies, Johannesburg, South Africa). For TEM, material was dehydrated using alcohol and propylene oxide and then embedded in Spurr's medium. A diamond or glass knife was used to cut silver sections that were mounted onto copper grids. A Jeol JEM 1011 transmission electron microscope (Advanced Laboratory Solutions, Johannesburg, South Africa) at 80 kV was used to provide detailed micrographs of spermatozoa for subsequent description. All images were captured digitally as either 'jpeg' or 'tiff' files.

### Sperm concentration and sperm motility

The contents of one or both ampullae were emptied into 10-20 μl Ham's F10 medium containing 3% bovine serum albumin at 28°C. Five micro litres of this sample was withdrawn using a micro pipette and a Leja "chamber" slide (20 μm deep and 5 μl volume) (Leja Products B.V., Nieuw Vennep, The Netherlands) was filled. The Leja slide was placed onto a temperature controlled stage of the Nikon E50i microscope (set at 28°C). A 10 × negative phase contrast objective in conjunction with a phase contrast condenser was used to study sperm motility by means of the Motility/Concentration module of the SCA system, version 4.1 (Microptic S.L., Barcelona, Spain) at 50 frames/second. The SCA system measures the percentage motility and eight kinematic parameters as indicated in Table 3. The SCA cut-off values for fast, medium and slow swimming spermatozoa were based on curvilinear velocity (VCL) = Fast > 45 > Medium > 35 > Slow. The SCA system also accurately determines the sperm concentration of a sample when using the above mentioned Leja slide (calibrated against a Neubauer hemacytometer).

**Table 3:** The different sperm kinematic parameters recorded with computer aided semen analysis (CASA)

Parameter	Unit	Description
Motility	%	Total motility
Concentration	×10^6^/ml	Number of spermatozoa
VCL	μm/s	Curvilinear velocity along actual swimming path
VSL	μm/s	Straight-line velocity along shortest path from start to end point
VAP	μm/s	Average path velocity based on every 5th frame of VCL path
LIN	%	Linearity of a curvilinear path, expressed as VSL/VCL
STR	%	Straightness, expressed as VSL/VAP
WOB	%	Wobble, expressed as VAP/VCL
ALH	μm	Amplitude of lateral head displacement
BCF	Hz	Beat cross frequency based on VCL crossing VAP per second

### Statistical analysis

MedCalc, Version 7 (Mariakerke, Belgium) was used for all statistical analyses. Descriptive statistics was used for calculation of averages and standard deviations (SD). Comparisons of sperm morphometry parameters were performed using either Anova or unpaired T-tests among the different groups and p < 0.05 was considered significant.

## Authors' contributions

All authors contributed to the study design, data sampling and data analyses. GvdH and LM collated the results and drafted the paper. All authors read and approved the final manuscript.
